# The precarious state of community engagement in African clinical trials: A systematic review of funding, workforce gaps, and the path to institutionalization

**DOI:** 10.1371/journal.pgph.0006484

**Published:** 2026-08-13

**Authors:** Luchuo Engelbert Bain, Sidney Sangong, Oluwaseun Adewunmi, Basile Njei

**Affiliations:** 1 African Population and Health Research Center (APHRC), Nairobi, Kenya; 2 Department of Psychology, Faculty of Humanities, University of Johannesburg, Johannesburg, Auckland Park, South Africa; 3 Centre Hospitalier de Châteauroux Le Blanc, Châteauroux, France; 4 Zentrum fur Medizin und Geselschaft, Department of Medical Anthropology, University of Freiburg, Freiburg, Germany; 5 Interfaculty Initiative in Planetary Health (IIPH), Nagasaki University, Nagasaki, Japan; 6 Section of Digestive Diseases, Department of Medicine, Yale University, New Haven, Connecticut, United States of America; 7 Engelhardt School of Global Health and Bioethics, Euclid University, Bangui, Central African Republic; Johns Hopkins University Bloomberg School of Public Health, UNITED STATES OF AMERICA

## Abstract

Community engagement (CE) is an ethical imperative for clinical trials, yet it is often treated as a peripheral activity rather than a core scientific requirement. In light of the Africa CDC’s mandate to achieve 60% local vaccine production by 2040, establishing robust community engagement frameworks within African clinical trials is vital. We systematically synthesized evidence on CE approaches, with a focus on funding and sustainability, utilizing the 2020 Preferred Reporting Items for Systematic Reviews and Meta-Analysis (PRISMA) checklist. Peer-reviewed research articles on CE in clinical trials across Africa published from January 2000 to February 2026 in English or French were retrieved from eight databases: MEDLINE, EMBASE, CINAHL, Cochrane Library, WHO Afro Library, African Journals Online databases, Scopus, Web of Science, and Global Health, alongside gray literature, reference lists, EDCTP reports, and Africa CDC documents. Study quality was appraised using the Mixed Methods Appraisal Tool (MMAT), and spatial analysis was performed using R with the sf and ggplot2 packages. Thirty studies met the inclusion criteria. Analysis revealed a dependency trap with approximately 90% of clinical trials in sub-Saharan Africa estimated to be funded by external international donors, creating a sustainability gap where essential trial components, such as community engagement and laboratory infrastructure, often dissolve upon the conclusion of a specific grant. We identified three primary typologies: instrumental (recruitment-focused), ethical (compliance-focused), and collaborative (partnership-focused). Findings show that while robust CE correlates with improved recruitment and social value, the reliance on uncompensated community labor and indirect funding models threatens the long-term viability of the African trial ecosystem. Transitioning from extractive CE to institutionalized CE is critical for Africa’s vaccine sovereignty, requiring funding models to shift toward direct, ring-fenced budgets to ensure pandemic preparedness and ethical integrity.

## Introduction

The United Nations consider community engagement as crucial for developing resilient, people-centered health systems that can ensure everyone has access to quality health services without financial hardship in accordance with the third Sustainable Development Goal [1]. Community engagement is defined as a collaborative process in which individuals, groups and organizations within a society work together in order to build steady relationships and enhance communication with the aim of achieving specific goals [2]. The key cornerstones for effective community engagement in health research include authenticity and credibility [3], with both local and global institutions acknowledging its importance in successful research. Regardless, the inequality in authority existing between researchers and community stakeholders causes community members to take the backseat in the decision-making process [4], often resulting in the withdrawal of community members due to a lack of trust [5].

Clinical trials are research studies conducted in humans to evaluate new or existing medical, surgical or behavioral interventions with the aim of assessing their safety and effectiveness [6]. Clinical trials are a critical part of health research assessing new methods of prevention, diagnosis, and treatment, incorporating new medications or devices for new surgical procedures, as well as modification of existing treatment algorithms [6].

Community engagement is a cornerstone of ethical and effective clinical trials, essential for building trust with community stakeholders, facilitating recruitment and ensuring equitable outcomes [5]. Moreover, in clinical trials, the community engagement (CE) process involves bidirectional communication to understand community needs and perspectives, which can lead to patient-centred studies, greater diversity in enrollment and improved patient retention [7].

In Africa, the stakes for CE have never been higher. As the continent advances its sovereignty agenda most notably the Africa CDC’s goal to produce 60% of vaccines locally by 2040 [8], the traditional top-down models of engagement are no longer fit for purpose. Historically, CE in Africa has been project-specific, often dictated by the requirements of Global North sponsors rather than local priorities [9]. This has resulted in a landscape that is fragmented, underfunded, and lacks a professionalized workforce.

While clinical trials are essential for evaluating new interventions [6], their success in Africa is increasingly dependent on navigating complex socio-political terrains. Current CE practices often focus on therapeutic misconception or recruitment metrics, yet rarely address the structural funding gaps that leave Community Advisory Boards (CABs) under-resourced and vulnerable to exploitation [5,7]. There is an urgent need to map the political economy of CE in Africa. This systematic review addresses this gap by asking:

- **Typology:** How is CE currently conceptualized is it transformative or merely instrumental?
- **The Funding Gap:** Who pays for CE, and how does the financing model influence its independence?
- **Human Capital:** Does a professional career pathway exist for CE practitioners in Africa?

The aim of this study was to systematically map, analyze, and synthesize evidence on community engagement (CE) in clinical trials across Africa, with particular focus on funding patterns, human resource capabilities, challenges, and opportunities for enhancing CE effectiveness and sustainability.

## Materials and methods

### Study design and search strategy

The 2020 Preferred Reporting Items for Systematic Reviews and Meta-Analysis (PRISMA) guidelines were employed [10]. In order to select suitable studies, A Boolean search string was developed using terms such as: “community engagement” or “community participation” or “community involvement” or “stakeholder engagement” or “community implication” or “community-based” or “public engagement” or “community advisory board” or “participatory research” in combination with the terms “clinical trial” or “clinical research” or “clinical study” or “vaccine trial” or “drug trial” or “intervention study” AND “Africa” then used to search a total of eight databases, namely MEDLINE, Scopus, Web of Science, CINAHL, and Global Health, Cochrane Library, African Journals online and WHO Afro Library. In order to capture the landscape of practice beyond peer-reviewed literature, we manually searched gray literature sources, including EDCTP annual reports and Africa CDC technical documents. The search encompassed literature published between January 1, 2000, and February 28, 2026.

### Study selection

The inclusion criteria for this review were published studies exploring community engagement in clinical trials in Africa. All included studies were either published in English or French. The inclusion criteria for this review were: peer-reviewed publications in English or French focused on community engagement in clinical trials, conducted in African countries/sub-Saharan Africa, high-quality grey literature addressing at least one of the research questions for review, and published between January 1 2000 and February 28 2026. The exclusion criteria were: studies with neither a specific focus on community engagement nor clinical trials, studies conducted before the year 2000, studies out of Africa, personal opinions, editorials, commentaries without empirical data, conference abstracts without full text and duplicate publications. By means of the eligibility criteria, the titles and abstracts of citations were screened prior to full-text assessments of selected articles for inclusion in this study. Duplicate records were removed using EndNote 20 [11], and the remaining citations were imported into Rayyan QCRI [12] for blinded screening. LEB and SS independently screened titles and abstracts, followed by a full-text review. Disagreements were resolved through consensus with OA and BN.

### Quality appraisal of included records

To ensure the reliability of our findings, we conducted a Quality Appraisal of all included studies using the Mixed Methods Appraisal Tool (MMAT) (2018 version) [13]. This tool allowed us to evaluate the methodological rigor of qualitative, quantitative, and mixed-methods studies simultaneously as illustrated in Annex 1 in S1 File. Each study was evaluated based on five design-specific criteria. Discrepancies in quality scores obtained by SS and LEB were resolved through a tie-breaker discussion with BN. Studies were not excluded based on quality scores; however, the weight of their contribution to the thematic synthesis was adjusted, with lower-quality studies (scoring < ***) being treated as supplementary rather than pivotal evidence.

### Data extraction and synthesis

Data were extracted into a standardized Excel matrix capturing: author, year, country, trial phase, CE typology, and specific data on funding and workforce. We employed Braun and Clarke’s Six-Phase Thematic Analysis [14]. This was an iterative, reflexive process where codes were generated inductively from the data and then mapped deductively against our research questions. This dual approach ensured that we captured both the state of the art and the underlying structural gaps in CE institutionalization.

### Geospatial visualization and software

To evaluate the geographic distribution and funding architectures of the included clinical trials, descriptive geospatial mapping and spatial visualizations were executed using R statistical software (version 4.5.3; R Foundation for Statistical Computing) [15]. Country-level aggregate data extracted from the National Institutes of Health (NIH) World RePORT database were programmatically joined with a high-resolution administrative boundary shapefile of the African continent, retrieved from the Natural Earth database via the rnaturalearth package (v1.0.1). Spatial data geometries were managed and projected utilizing the Simple Features standard via the sf package. To visually represent trial density, continuous choropleth maps were constructed using the ggplot2 framework. Color scales were mapped using the perceptually uniform and colorblind-friendly viridis palette to ensure visual accessibility and reduce bias in density interpretation. To facilitate comparative analysis across distinct funding organizations, multi-panel faceted maps were generated. Final figure dimensions, resolutions, and text scaling were systematically optimized to maintain legibility and graphic integrity across all panels. In accordance with open-science and reproducibility principles, the fully annotated R script, underlying spatial datasets, and source NIH World RePORT extraction files are hosted and publicly available in the Supporting Information files. The PRISMA checklist for this study is as well available in the supporting information files.

### Expected outcome and impact

This review generated actionable evidence to guide funders, policymakers, and clinical trial sponsors in strengthening CE funding, workforce capabilities, and accountability systems, laying the foundation for an equitable, community-anchored clinical trials ecosystem in Africa.

## Results

### Search results

**Fig 1:**
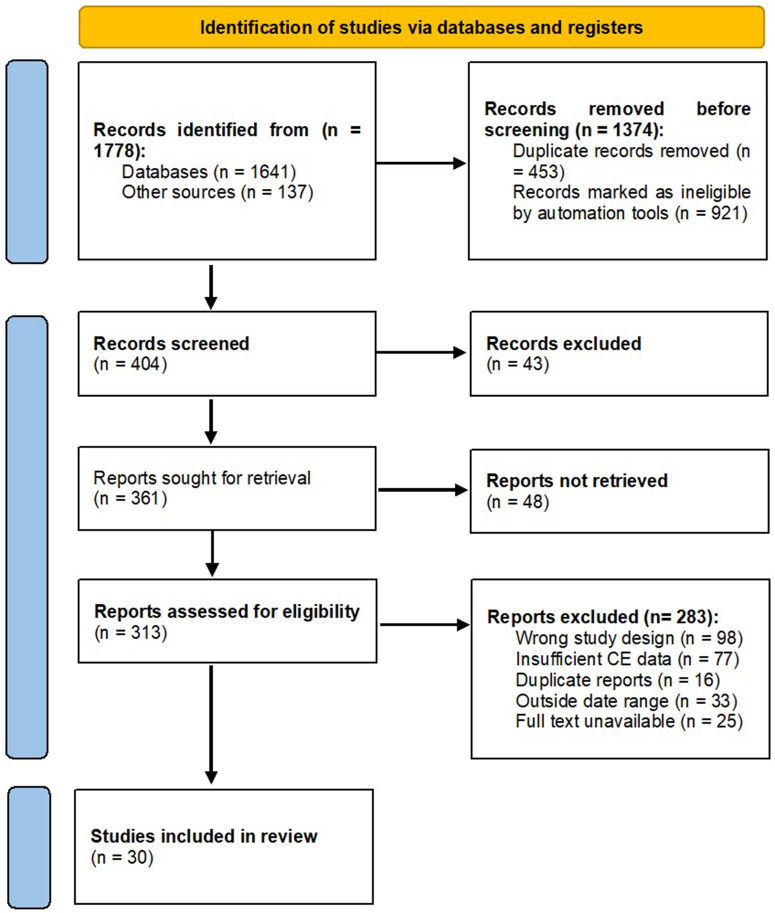
PRISMA Flow Diagram of the study selection process.

After database search, 1778 records were initially identified through search from the seven databases, more precisely: MEDLINE (450), Scopus (380), CINAHL (180), Web of Science (97), WHO afro library (152), African journals online databases (96) and Global Health (210). Moreover, 137 additional records were retrieved from other sources, including: Grey literature (27), Reference lists (36), EDCTP reports (26), and Africa CDC documents (18). After screening the titles alongside their abstracts, 313 were retained. Following a full-text eligibility evaluation of these 313 studies, 30 met the inclusion criteria for our study. Fig 1 illustrates the PRISMA flow process for the selection of this review Fig 1.

**Fig 2:**
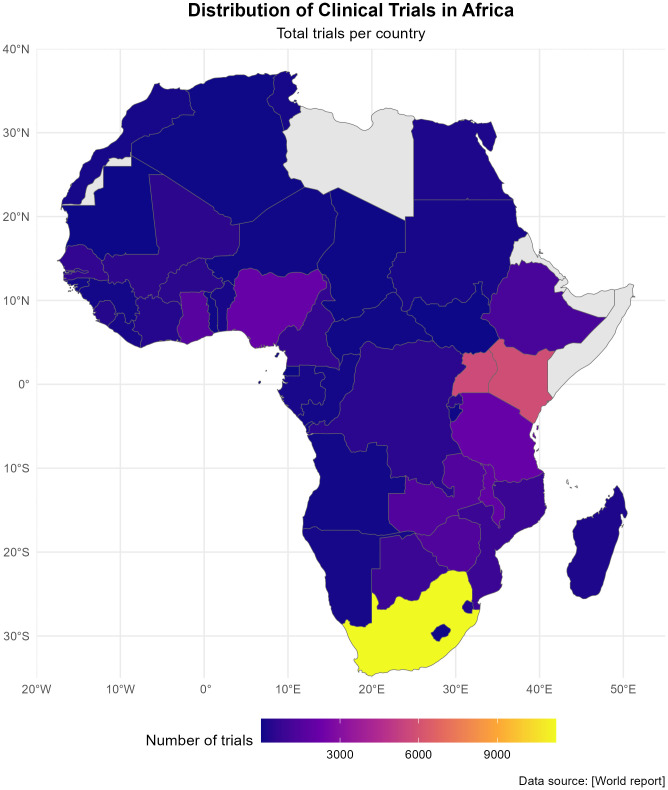
Distribution of Clinical trials across Africa produced using R on the Natural Earth Public domain (http://www.naturalearthdata.com/about/terms-of-use/) with African Clinical trials data obtained from the NIH World RePort.

**Fig 3:**
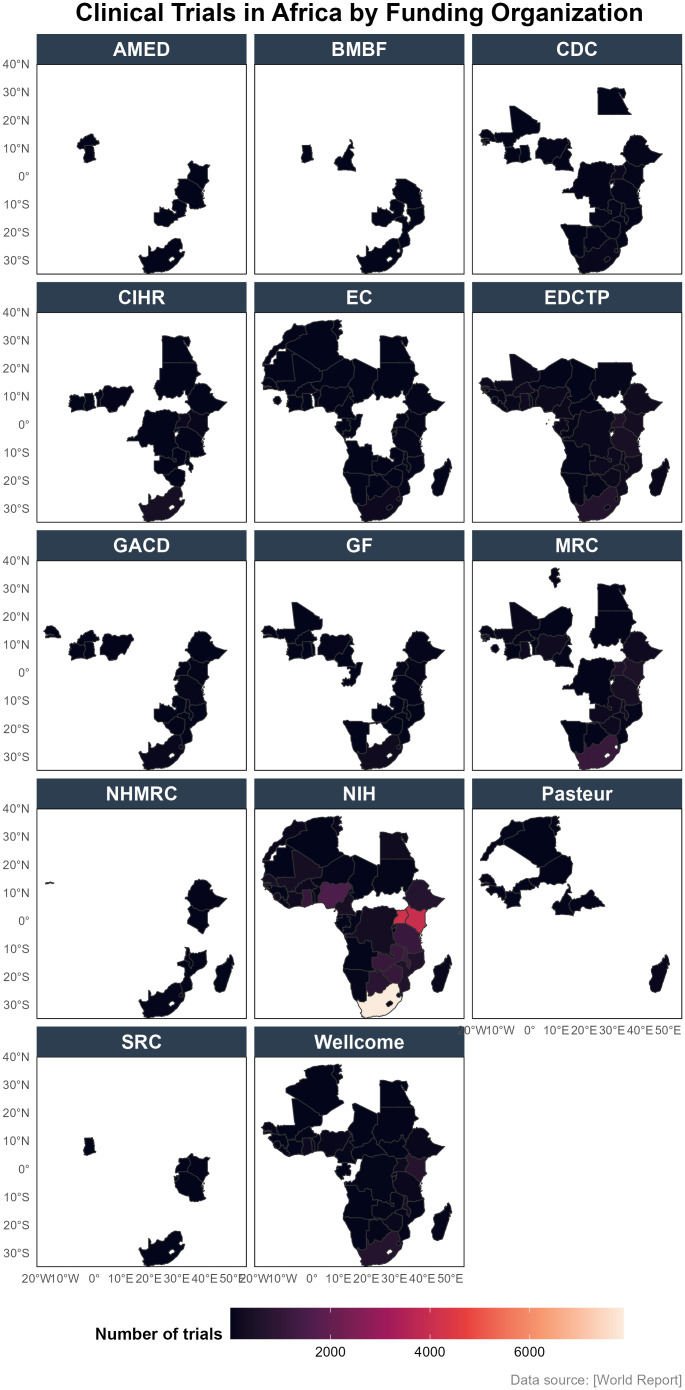
Distribution of Clinical trials by funding organizations across Africa produced using R on the Natural Earth Public domain (http://www.naturalearthdata.com/about/terms-of-use/) with African Clinical trials data obtained from the NIH World RePort.

### Overview of included studies

All thirty included records were conducted between 2008 and 2025. Eight of the included articles were qualitative studies based mostly on in-depth interviews and to a lesser degree, focused group discussions; meanwhile, five of these were quantitative, three mixed studies and two systematic reviews. Ten out of the thirty selected studies were single-country studies, including South Africa (1), Ghana (2), Congo (1), Uganda (3), Tanzania (1), Kenya (1) and Malawi (1). Twenty out of the thirty included studies were multi-country studies, including: sub-Saharan Africa (6), all member states of the African union (11), West Africa as a whole (1), a multi-country study conducted in Burkina Faso, Mali and Tanzania (1) and finally a multi-country study conducted in Thailand, India, South Africa and Canada. South Africa. Categorization of the thirty included studies revealed a distinct thematic distribution: more precisely, seven studies [16–22] (23.3%) addressed vaccine-related interventions, whereas twenty three [9,23–42] 76.7% comprised clinical trials targeting infectious disease therapeutics.

Table 1 shows the thematic results of the review from the thirty included records. Eight out of the thirty records selected for this review provided details on the current state of community engagement in clinical trials in Africa [8,11–17]. Eight of the thirty included manuscripts in this review examined the typology of community engagement initiatives in clinical trials in Africa [8,11,12,17–21]. Additionally, six out of the thirty included articles expounded on the funding patterns of community engagement activities in clinical trials in Africa [16,22–26]. The level of human resource capacity for CE in clinical trials in Africa was explored by five records included in this review [27–31]. Regarding barriers to effective CE implementation in clinical trials in Africa, this was explored by seven studies in the review [9,16,20,23,30,34,38]. While barriers to effective CE in monitoring and evaluation were reported by three manuscripts [20,33,39]. Pertaining to potential future opportunities to strengthen CE for clinical trials, in pandemic and epidemic preparedness, this was detailed by five included records of the systematic review [34–38].

**Table 1:** Themes and Sub-themes of the qualitative review.

The Current State of Community Engagement in Africa		
Themes	Sub-themes	Research Papers
Progress and Drivers	Trust Building	Bain *et al*, (2022) Nakibinge *et al*, (2009) Sangong *et al*, (2025)
	Reaction to ethical issues	Lawrence *et al*, (2016) Manda-Taylor *et al*, (2013) Baron *et al*, (2018) Graef *et al*, (2020)
	Incorporating major Research Initiatives including: Pandemic preparedness trials, Genomics, HIV Prevention trials and the International AIDS Vaccine Initiatives	Newman *et al*, (2015) Nakibinge *et al*, (2009)
**Typology of Community Engagement Initiatives in Africa**		
Involvement of Community stakeholders per standard community engagement Framework	Inform the community prior to research	Folayan *et al*, (2019) Nyika *et al*, (2010) Nakibinge *et al*, (2009) Gobat *et al*, (2025) Folayan *et al*, (2021) Bain *et al*, (2022) Sangong *et al*, (2025) Newman *et al*, (2015)
	Consult the community and have their takes and concerns	Folayan *et al*, (2019) Nyika *et al*, (2010) Nakibinge *et al*, (2009) Gobat *et al*, (2025) Folayan *et al*, (2021) Bain *et al*, (2022) Sangong *et al*, (2025) Newman *et al*, (2015)
	Collaborate with community stakeholders throughout the research process	Folayan *et al*, (2019) Nyika *et al*, (2010) Nakibinge *et al*, (2009) Gobat *et al*, (2025) Folayan *et al*, (2021) Bain *et al*, (2022) Sangong *et al*, (2025)
	Partnership	Folayan *et al*, (2019) Nyika *et al*, (2010) Nakibinge *et al*, (2009) Gobat *et al*, (2025) Folayan *et al*, (2021) Bain *et al*, (2022) Sangong *et al*, (2025)
	Community-led Empowerment	Folayan *et al*, (2019) Nyika *et al*, (2010) Nakibinge *et al*, (2009) Gobat *et al*, (2025) Folayan *et al*, (2021) Bain *et al*, (2022) Sangong *et al*, (2025) Newman *et al*, (2015)
**Funding Patterns of Community Engagement Activities in Clinical Trials in Africa**		
**Themes**	**Sub-themes**	**Research Papers**
Direct Grant Funding from External Sponsors	Pharmaceutical and Biotech Companies	Marsh *et al*., (2008) Graef *et al*, (2020)
	International Funding bodies including: Bill & Melinda Gates Foundation, the National Institutes of Health (NIH) in the US, and the Wellcome Trust	Marsh *et al*., (2008) Graef *et al*, (2020)
Institutional and Donor-Specific Funding	The European & Developing Countries Clinical Trials Partnership (EDCTP)	EDCTP, (2021)
	Research Institutions’ Overheads	EDCTP, (2021)
Indirect Funding model	Volunteering	Pancras *et al*, (2022) Mijumbi *et al*, (2023) Reddy *et al*, (2010)
	Funding from existing Public Health Infrastructures	Pancras *et al*, (2022) Mijumbi *et al*, (2023) Reddy *et al*, (2010)
**Level of human resource capacity for CE in clinical trials in Africa**		
**Themes**	**Sub-themes**	**Research Papers**
Skills	Mixed Skill Sets	Tindana *et al*, (2020)
	Lack of Standardized Training	Yakubu et al, (2018)
	Critical Skill Gaps	Tindana *et al*, (2020)
Career Pathways	Job Titles and Descriptions	The Global Health Network, (2017)
	Limited Advancement Opportunities	The Global Health Network, (2017)
	Lack of Professional Recognition	The Global Health Network, (2017)
Institutional support	Variable Commitment	Pratt *et al*, (2018)
	Insufficient and Insecure Funding	Pratt *et al*, (2020)
	Emerging Frameworks and Infrastructure	Pratt *et al*, (2020)
**Barriers to effective CE Implementation in Africa**		
**Themes**	**Sub-themes**	**Research articles**
Systemic and Infrastructural Barriers	Underfunding and Short-term Planning	Bain *et al*, (2022) Sangong *et al*, (2025) Baron *et al*, (2018) Tindana *et al*, (2011)
	Weak Regulatory Frameworks	Bain *et al*, (2022) Sangong *et al*, (2025) Tindana *et al*, (2011)
	Limited Local Research Capacity	Bain *et al*, (2022) Sangong *et al*, (2025) Tindana *et al*, (2011)
Socio-Cultural and Historical Context	Legacy of Exploitation and Mistrust	Bain *et al*, (2022) Newman *et al*, (2015) Sangong *et al*, (2025) Yakubu *et al*, (2018)
	Complex Community Hierarchies and Power Dynamics	Bain *et al*, (2022) Newman *et al*, (2015) Sangong *et al*, (2025) Marsh *et al*, (2008)
	Therapeutic Misconception	Bain *et al*, (2022) Newman *et al*, (2015) Sangong *et al*, (2025)
Communication and Literacy Barriers	Linguistic Diversity and Conceptual Translation	Bain *et al*, (2022) Newman *et al*, (2015) Sangong *et al*, (2025)
	Low Health and Scientific Literacy	Bain *et al*, (2022) Newman *et al*, (2015) Sangong *et al*, (2025)
**Barriers to effective CE in Monitoring and Evaluation in Africa**		
**Themes**	**Sub-themes**	**Research articles**
Quantifying accomplishments	Lack of Standardized Metrics	Baron *et al*, (2018) Clinical Trials Transformation Initiative (CTTI), (2015)
	Qualitative vs. Quantitative Data	Clinical Trials Transformation Initiative (CTTI), (2015)
Methodological and Logistical Hurdles	Cost and Time Intensity	Clinical Trials Transformation Initiative (CTTI), (2015)
	Attribution vs. Contribution	Clinical Trials Transformation Initiative (CTTI), (2015)
Power Dynamics in Feedback Loops	Lack of Transparent Feedback Mechanisms	Tindana *et al*, (2020)
	Community Capacity to Participate in M&E	Tindana *et al*, (2020)
**Potential future opportunities to strengthen CE for clinical trials, in pandemic and epidemic preparedness**		
**Themes**	**Sub-themes**	**Research articles**
	Incorporating CE into National Public Health Infrastructure	Africa CDC (2023)
	Co-creation	Africa CDC (2023)
	Leverage and Strengthen Local Leadership and Trusted Messengers	World Health Organization, (2019)
	Invest in Sustainable Local Research Capacity and Ownership	Baker *et al*, (2025)
	Develop Dynamic, Multi-Platform, and Two-Way Communication Strategies	Asante *et al*, (2013)
	Ensure Post-Trial Access and Clear Benefit-Sharing Agreements	CIOMS (2016)

### The current state of community engagement in Africa

Community Engagement (CE) in African clinical trials is increasingly shifting from a marginal, ethics-focused formality to a core, strategic element of research. This change is motivated by historical ethical missteps, the complexities of emerging research areas like genomics and pandemic preparedness, and a growing acknowledgement of CE’s scientific and ethical importance. Despite this progress, CE practices are still inconsistent, frequently underfunded, and struggle to evolve from basic consultation toward authentic, collaborative partnerships.

Regarding the theme of progress and drivers in the current state of CE approaches in clinical trials in Africa, three sub-themes were disclosed during our review, including: trust building, adapting to ethical hurdles, and incorporation of major Research Initiatives such as pandemic preparedness trials, Genomics, HIV Prevention trials and the International AIDS Vaccine Initiatives. Pertaining to the trust-building sub-theme in the current state of CE approaches in clinical trials in Africa, three studies in this review expounded on this [9,17,23]. All three studies elaborated on the fact that building trust has been the cornerstone of effective community engagement, yet this trust has been fragile and most often eroded by historical exploitation or negative research experiences [9,23]. Moreover, in order to successfully establish and maintain this trust, it was critical to move beyond one-off interactions and instead foster sustained, respectful, and two-way partnerships that involved local stakeholders from the outset and include genuine capacity-building [17]. Ultimately, a long-term commitment that promotes co-ownership of the research helps ensure communities feel the work was being done with and for them, rather than on them [17]. Concerning reaction to ethical issues as the second sub-theme under this category, we explored four studies in this review [13–16]. These studies revealed that the ethical challenges within community engagement are often deeply structural, rooted in how Community Advisory Boards (CABs) are constituted, funded, and institutionally supported, which directly impacts their ability to address ethical dilemmas [25]. While formal frameworks like Good Participatory Practice (GPP) provide valuable structure, leading to improved consent processes, rumor management, and trust, their success depended on real, long-term investment in training, funding, and institutional recognition to empower CABs [20,26]. Ultimately, for ethical intentions to become practice, the design of community engagement needs to be context-specific, tailoring who sits on CABs and how engagement occurs to local cultural, social, and resource realities [13–16]. The third and final sub-theme under this section was the incorporation of major research initiatives, such as pandemic preparedness trials, genomics, HIV Prevention trials and the International AIDS Vaccine Initiatives, reported by two studies in this review [16,17]. HIV research, particularly through long-term cohorts and prevention trials, has been the pioneering force for structured community engagement in Africa, establishing the necessity of continuous, well-resourced, and ethically sound models [16,17]. This foundational approach has now set a precedent, directly influencing engagement standards for newer fields like pandemic preparedness and genomics, which require similar foundations of trust, scientific literacy, and transparency [16]. Furthermore, large-scale international initiatives have demonstrated that substantial, coordinated funding can professionalize community engagement, creating consistent and effective frameworks that are being adopted across diverse areas of research [16,17].

### The typology of community engagement initiatives in clinical trials in Africa

With regards to the typology of community engagement initiatives in clinical trials in Africa, eight of the thirty included studies in this review provided insights into this [8,11,12,17–21]. Overall, Community engagement (CE) in African clinical trials has been conceptualized along a continuum of power-sharing, often aligned with the International Association for Public Participation (IAP2) spectrum [43]. At the lower end, CE activities are focused on unidirectional communication, which entails informing or consulting the community stakeholders. The more advanced modalities labelled “Involve” and “Collaborate” entail iterative dialogue, co-development of study materials, and shared participation in governance processes [45]. The highest tier, which has to do with empowerment in which communities assume substantive decision-making authority, is seldom achieved within the constraints of regulated clinical trial environments but remains an aspirational benchmark for equitable researcher community partnerships [8,12,17–19,21]. Although contemporary practice has gradually been shifting from minimal consultation toward more substantive involvement, progress is uneven and frequently limited by structural barriers, including insufficient funding and weak institutional support for sustained engagement [8,11,12,17–21].

### Funding patterns of community engagement activities in clinical trials in Africa

The geographic distribution of clinical trials across Africa showed substantial heterogeneity (Annex 2 in Fig 2). Countries with the highest trial density included: South Africa with over 9000 trials and Kenya with over 6,000 trials, while several nations had minimal (below 3,000 trials) and finally countries like Somalia, Djibouti, Eritrea and Libya with no recorded trials. When stratified by funding organization, distinct geographic patterns emerged (Annex 3 in Fig 3). For instance, the current landscape of clinical trial funding in Africa is characterized by a Southern-East Axis of investment, where South Africa, Kenya, and Uganda serve as the primary focal points for nearly all major global health actors. Further highlighting a critical funding gap of the northern, western and central axes. Moreover, this geographic concentration is driven by a two-tiered funding approach: high-volume, continental reach from organizations like the National Institutes of Health (NIH) and Wellcome Trust, which maintain the most extensive footprints and highest trial densities contrasted with the regional specialization of groups like Pasteur in Francophone West Africa or the Medical Research Council (MRC-UK) in East Africa. While pan-African initiatives from the European and Developing Countries Clinical Trials Partnership (EDCTP) and the European Commission (EC) provide broad geographic coverage. Moreover, smaller funders such as the Japan Agency for Medical Research and Development (AMED) and the Federal Ministry of Education and Research (BMBF-Germany) operate through a hub-and-spoke model, further reinforcing the dominance of established research infrastructures and National Public Health Institutes in specific regional centers. In a nutshell, the spatial analysis of these funding patterns reveals a monolithic reliance on non-African organizations. This lack of domestic or regional investment creates a fragile funding ecosystem where African clinical research capacity is contingent upon external institutional stability and donor-driven objectives.

Overall, this review uncovered three themes and six sub-themes pertaining to the funding patterns of CE activities in clinical trials in Africa reported by six included records [16, 22–26]. Direct grant funding from external sponsors was the first theme under this section, from which two sub-themes were: Pharmaceutical and Biotech Companies and International Funding bodies, including Bill & Melinda Gates Foundation, the National Institutes of Health (NIH) in the US, and the Wellcome Trust. Each of these subthemes was reported by two studies from the review, respectively [28,30]. Operational and empirical evidence consistently highlighted the chronic underfunding of community engagement (CE) in African clinical trials by commercial sponsors. Graef et al. (2020) observed that despite CE’s critical role in ethical conduct, recruitment, and trust-building, pharmaceutical and biotechnology sponsors frequently fail to designate sufficient, dedicated budgets for it [28]. This is compounded by findings that without explicit financial commitments from external funders, including industry partners, research institutions cannot maintain substantive engagement when the need arises [30]. Collectively, this evidence positions CE as a peripheral cost rather than a core infrastructural requirement and advocates for consistent, ring-fenced funding from industry to enable effective, context-appropriate engagement [28,30]. The second sub-theme under direct grant funding from external sponsors covered by the same dual previously mentioned [28,30] was funding from international funding bodies such as the Bill & Melinda Gates Foundation, the National Institutes of Health (NIH) in the US, and the Wellcome Trust. Sustainable community engagement in African clinical research has been underpinned by dedicated, institutional-level funding from major international donors, which enabled long-term infrastructure rather than fragmented, project-specific activities [28,30]. For instance, funders like the NIH and the Wellcome Trust directly supported embedded networks, training, and structured dialogue within research consortia and long-standing programmes, fostering robust, trust-based relationships [28]. Complementarily, the Gates Foundation contributes indirectly by investing in systemic capacity, such as clinical trial platforms and registries, which enhance the overall infrastructure for transparent and inclusive community interactions [28]. Collectively, these models demonstrate that strategic, sustained investment was fundamental to transitioning community engagement activity to an integral component of the research ecosystem [28,30].

Secondly, institutional and donor-specific funding was another theme under this section, covering the two sub-themes: The European & Developing Countries Clinical Trials Partnership (EDCTP) and Research Institutions’ Overheads, all reported by one record in the review [32]. The European and Developing Countries Clinical Trials Partnership (EDCTP) strategically fosters and embeds Community Engagement (CE) through a multi-pronged approach. This includes direct financial support for project-specific CE activities, facilitation of deliberative dialogues to address local ethical considerations, and the execution of stakeholder workshops aimed at building ethics and regulatory capacity. To ensure the long-term viability of these efforts, a dedicated working group has been established to systematically review all research protocols through a CE framework and to develop comprehensive strategies for its successor program, EDCTP. The latter will feature a focused agenda on late-stage clinical trials, research involving vulnerable populations, and studies concerning emerging infectious diseases [32].

The indirect funding model for CE activities in clinical trials in Africa was the final theme in this section, reported by three studies [24–26], which disclosed two sub-themes, including: volunteering and funding from existing Public Health Infrastructures. Evidence from these three studies conducted in Tanzania, Uganda, and South Africa consistently indicates that the reliance on volunteering as an indirect funding mechanism for Community Advisory Boards (CABs) is both operationally unsustainable and ethically questionable [24–26]. This model contributes to financial strain, burnout, and elevated turnover rates among uncompensated CAB members. Consequently, this practice risks the exploitation of community altruism, compromises the quality and fidelity of community engagement (CE), and can introduce a conflict of independence [21]. Specifically, dependence on research institutions for intermittent stipends may inhibit CAB members from providing essential critical oversight [31]. These findings collectively establish that achieving effective and ethical CE requires formal, direct funding to appropriately remunerate members for their dedicated time and expertise [24–26]. Funding from existing Public Health Infrastructures was the final sub-theme in this section, which covered three studies [21,24,31]. The collective evidence derived from these studies indicates that the strategic reliance upon existing Public Health Infrastructures (PHI) as an indirect mechanism for funding Community Advisory Boards (CABs) is operationally inefficient and structurally unsustainable [21,24,31]. While this approach benefits from the use of established institutional systems, it frequently results in ambiguity of roles, places an unacceptable burden on PHI personnel, and generates critical conflicts between routine service provision and research-specific engagement activities. This model effectively constitutes an unfunded mandate for the public sector, which ultimately jeopardizes the effectiveness, impartiality, and long-term viability of the CAB structure.

### The level of human resource capacity for CE in clinical trials in Africa

Current assessments suggest that the human resource infrastructure supporting Community Engagement (CE) in clinical trials across Africa is at a nascent to intermediate stage of maturity. While there is an increasing consensus regarding the pivotal role of CE, this conceptual evolution has not been sufficiently matched by the development of robust, standardized, and adequately supported human resource systems. Three themes were disclosed in this section of the systematic review covered by five studies [27–31]. Pertaining to the skill set as the first theme in this section, the necessity for effective Community Engagement (CE) inherently demands a sophisticated interdisciplinary competency profile, integrating knowledge domains from scientific, social science, and communication disciplines. However, analysis of current practice identifies significant systemic weaknesses in its execution. The existing CE workforce often exhibits mixed skill sets because community-sourced staff, while offering invaluable cultural and linguistic capital, frequently lack formal credentials in research ethics, regulatory frameworks, and clinical methodology [33]. Conversely, core clinical trial staff commonly lack adequate training in qualitative research methods, cultural sensitivity, and effective participatory techniques [33]. A fundamental challenge contributing to this is the systemic absence of standardized, accredited training programs for CE professionals in African research environments [34]. The prevalent model relies instead on informal, training and workshops, which leads directly to variable quality control and inconsistent professional capability [34]. This capacity deficit is most critical in areas essential for effective liaison, encompassing the skills required for Scientific Advocacy, Ethical Negotiation, Conflict Resolution by mitigating historical mistrust, and Systematic Monitoring and Evaluation [33].

The next theme examined under this section expounded on the career pathways of community members explored by one record in this review [35]. The underdeveloped career structures for Community Engagement (CE) professionals present a major impediment to the attraction and retention of skilled human resources. This structural deficiency is characterized by several key features. Firstly, there is an ambiguity in role definition because job titles and descriptions for CE personnel, such as Community Liaison Officer or Engagement Specialist, lack standardization across diverse research institutions and funding agencies [35]. This ambiguity contributes to role confusion and results in the undervaluation of the specialized professionalism necessary for effective CE execution. Secondly, CE is frequently conceptualized as a support or ancillary function rather than a strategic professional pathway, resulting in constrained advancement trajectories. Consequently, there is a scarcity of clearly defined hierarchical pathways for advancement into senior management or leadership roles within host research institutions [35]. This absence of a viable career trajectory contributes directly to elevated personnel turnover, as skilled staff migrate to sectors offering superior professional prospects. Finally, the field suffers from a deficiency in professional credentialing because, unlike established domains such as data management or laboratory science, CE in clinical research lacks widely recognized professional certifications or accrediting bodies [35]. This absence reinforces the perception of CE as a soft skill rather than a specialized, professional discipline.

Regarding institutional support as the final theme in this category, three sub-themes were disclosed, explored by two studies in this review [36,37]. The institutional ecosystem essential for fostering CE competencies and professional progression exhibits marked instability. While institutional commitment is paramount, it currently demonstrates high variability: CE support is often determined by PI discretion or extrinsic funding mandates, rather than being universally embedded as a core institutional imperative [36]. Concurrently, CE budgeting remains inadequate and structurally insecure. Salaries for CE staff are typically constrained by the duration of project grants [37], thereby generating job insecurity and undermining the ability to maintain sustained engagement continuity. Notwithstanding these structural deficiencies, the development of formal frameworks and dedicated infrastructure by entities such as the Africa CDC, EDCTP, and institutional models like KEMRI-Wellcome Trust and AHRI represents a positive trend toward the professionalization and stabilization of CE support [36,37].

### Barriers to effective CE implementation in Africa

Regarding barriers to effective CE implementation in clinical trials in Africa, this was explored by seven studies in the review [9,16,20,23,30,34,38], covering three themes, namely: Systemic and infrastructural barriers, socio-cultural and historical context, and communication and literacy barriers. These themes were further divided into eight sub-themes.

With respect to the theme of systemic and infrastructural barriers, firstly, in regard to the sub-theme of underfunding and short-term planning, four studies [9,20,23,38] explored this, stating that: the implementation of meaningful community engagement (CE) in African clinical research was systematically hampered by insufficient financial investment and transient planning horizons [9]. Empirical evidence, including systematic reviews and case studies, indicated that CE was frequently compartmentalized as an ancillary function, which resulted in erratic budget allocations and discontinuous execution [9,23]. This financial instability precluded the development of robust engagement infrastructures, curtailed opportunities for sustained trust-building dialogue, and obstructed the formation of long-term partnerships with diverse community and traditional leadership systems [20,38]. Consequently, even in contexts where proven CE frameworks demonstrated efficacy, their application was compromised by a lack of dedicated, institutionalized funding. These factors collectively represented systemic barriers that transmuted CE from a continuous, ethically-grounded process into a series of episodic and largely symbolic interactions. Furthermore, three studies [9,23,38] disclosed structural barriers to effective CE concerning weak regulatory frameworks, noting that: the lack of robust and uniformly applied regulatory frameworks represented a major impediment to effective Community Engagement (CE) across African clinical trial settings. Empirical data from regional analyses indicated a widespread deficit in formal national and institutional CE mandates, resulting in substantial heterogeneity of practice [9,23]. Consequently, the quality and scope of engagement were often contingent upon project-specific priorities dictated by investigators or funders. This fragmentation was particularly acute in multisectoral research areas like One Health, where the absence of coordinated governance undermined structural planning [23]. Moreover, in the absence of formalized oversight, the tendency for researchers to engage in informal, unscripted interactions with local traditional leadership introduced operational instability, manifesting as ambiguous role definitions, undefined mutual expectations, and unclear lines of authority [38]. These governance failures ultimately limited transparency, eroded accountability, and obstructed the embedding of sustainable, ethically warranted community partnerships essential for high-fidelity research execution [9,23,38]. Limited local research capacity emerged as a critical systemic barrier to the effective operationalization of community engagement (CE) across African clinical research settings. Evidence from systematic reviews and regional case studies demonstrated that many institutions lacked personnel with specialized training in CE methodologies, social science, or cross-sectoral coordination, which resulted in inconsistent and often superficial engagement practices [9,23]. In multidisciplinary fields such as One Health, these capacity deficits were compounded by weak institutional linkages across the human, animal, and environmental health sectors, which undermined the development of integrated and sustained engagement strategies [23]. Case studies further illustrated that insufficient training, logistical resources, and institutional support constrained researchers’ ability to navigate complex community governance structures and collaborate effectively with traditional authorities [38]. Collectively, these limitations eroded the quality, continuity, and cultural grounding of CE, ultimately diminishing its effectiveness in supporting ethical and context-responsive clinical research in Africa.

In Regards to the theme of socio-cultural and historical context as a barrier to effective CE implementation in Africa, this was explored by five studies in this review [9,16,23,30,34], revealing three sub-themes including: legacy of exploitation and mistrust, complex community hierarchies and power dynamics, and therapeutic misconception. A legacy of historical transgressions in medical research had engendered profound and persistent mistrust, which continued to shape community responses to clinical trials across Africa [9,23]. High-profile cases, such as the Pfizer Trovan trial in Kano, Nigeria, crystallized a perception that research disproportionately allocated risks to host communities while channeling primary benefits to external actors [34]. These concerns were exacerbated by complex, multi-tiered social hierarchies, wherein reliance solely on formal leadership structures, such as chiefs or religious authorities, proved insufficient for representing the full spectrum of community interests [16,30]. This approach often obfuscated the perspectives of marginalized groups, including women, youth, and lower socio-economic strata, resulting in engagement that was superficial rather than inclusive [9,23]. Furthermore, therapeutic misconception remained a pervasive challenge, as many prospective participants equated research participation with guaranteed access to superior therapeutic care [9,23]. This fundamental misunderstanding compromised the integrity of the informed consent process and seeded disillusionment when trial outcomes diverged from expectations. Collectively, these factors constituted deeply rooted socio-historical challenges that were identified as critical to address for the attainment of ethically robust and contextually grounded community engagement in African clinical research.

Pertaining to the theme of socio-cultural and historical context as a barrier to effective CE implementation in Africa, this was explored by three studies [9,16,23] in this review, covering two sub-themes, namely: Linguistic Diversity and Conceptual Translation, and Low Health and Scientific Literacy. Linguistic heterogeneity across African research settings posed significant challenges for accurately conveying complex scientific concepts such as randomization, placebo control, and risk [9,23]. Translational adjustments intended to enhance comprehension often resulted in conceptual drift, limiting participants’ understanding of key trial elements [16]. These challenges were further exacerbated by generally low levels of health and scientific literacy, which restricted the capacity of potential participants to engage meaningfully with study information and complicated the attainment of valid informed consent [9,16,23]. Addressing these limitations required the development of innovative, context-sensitive communication strategies that more effectively bridged scientific terminology and local knowledge systems.

### Barriers to effective CE in monitoring and evaluation in Africa

Defining and evaluating the effectiveness of community engagement remained challenging due to the absence of standardized and validated metrics. There was no consensus on whether indicators such as recruitment efficiency, participant retention, or favorable community perceptions constituted meaningful measures of successful engagement, which limited the ability to monitor progress, compare approaches across studies, and ensure accountability [20,39]. Furthermore, many of the most substantive outcomes of community engagement, such as trust, empowerment, and the development of mutually respectful relationships, were inherently qualitative and thus difficult to operationalize within formal evaluation frameworks [39]. As a result, assessments often relied heavily on quantitative proxies, including the number of meetings conducted or participants in attendance, which failed to capture the depth, relational quality, or ethical significance of engagement processes [39].

Methodological and logistical constraints further impeded the rigorous monitoring and evaluation of community engagement activities. Comprehensive evaluation processes required substantial investments in data collection, analysis, and interpretation; however, in resource-limited settings, such commitments were often perceived as prohibitively costly and time-consuming, resulting in superficial or incomplete monitoring efforts [39]. Additionally, attributing specific trial outcomes—such as high participant retention or improved adherence—directly to particular engagement activities proved challenging, as these outcomes were influenced by multiple contextual factors, including healthcare quality, transportation barriers, and participants’ experiences with trial procedures and side effects [39]. This difficulty in distinguishing attribution from broader contributions limited the ability to demonstrate the distinct value of community engagement to funders and decision-makers.

Power imbalances within feedback processes further constrained the effectiveness of monitoring and evaluation of community engagement. Although community input was often solicited, transparent mechanisms for integrating this feedback into revisions of engagement strategies or trial procedures were frequently absent, which made the engagement process appear performative rather than genuinely participatory [33]. Moreover, meaningful involvement of community members in evaluative activities was limited by disparities in training, confidence, and institutional power. Many communities lacked the requisite skills or empowerment to critically assess engagement practices or to provide structured feedback to research teams, particularly when these teams were affiliated with large, well-resourced, and often international institutions [33]. These asymmetries curtailed the potential for feedback loops to function as equitable, bidirectional processes capable of strengthening engagement quality.

### Potential future opportunities to strengthen CE for clinical trials, in pandemic and epidemic preparedness

The efficacy of responses during public health crises was historically constrained by the episodic and non-systemic implementation of Community Engagement (CE). To enhance preparedness and coordination, a structural shift is required: CE activities must be institutionalized within the established national and regional public health governance structure, moving beyond reliance on temporary, externally funded projects [29]. A key recommendation proposes the creation and dedicated resourcing of standing CE entities, staffed by local expertise, and situated within Ministries of Health and their respective NPHIs throughout Africa. This institutional detachment from trial-specific funding ensures the cultivation of enduring community partnerships and the development of resilient communication channels for swift activation during emergencies [29]. The strategic integration of CE into routine services (e.g., vaccination and primary care) builds antecedent community trust, guaranteeing that established, authoritative community representatives are available to support the ethical conduct and operational demands of clinical trials during emergency research.

Implementing a co-creation model across all phases of the research process was identified as a key strategy for strengthening community engagement [29]. Unlike conventional approaches that sought community input only after pivotal design decisions had been made, co-creation involved Community Advisory Boards and local civil society organizations as active partners from protocol development through dissemination [29]. Their early and continuous participation ensured that study procedures were culturally and contextually appropriate, addressed locally relevant priorities, and were operationally feasible [29]. Community insights also informed practical adaptations such as aligning visit schedules with agricultural seasons or incorporating mobile clinics to enhance accessibility, recruitment, and retention.

During epidemics, reliance on institutional messaging proved insufficient for countering misinformation, highlighting the need to engage trusted local actors [18]. Individuals such as religious leaders, educators, youth group facilitators, and traditional healers were identified, trained, and supported to communicate scientific concepts in culturally and linguistically appropriate ways [18]. Their involvement enhanced credibility, facilitated accurate information dissemination, and strengthened community adherence to public health measures and research participation.

The prevalence of “parachute research” in African clinical trials highlighted the need for sustainable local capacity building. Investments in training African researchers, ethics committee members, and community liaison officers, combined with African leadership in trial design and shared data ownership, enhanced local ownership and community trust [40]. This approach not only increased participation and engagement but also developed a skilled workforce capable of supporting future research and epidemic responses.

Static communication methods were insufficient for effective community engagement, necessitating dynamic, multi-platform, and two-way strategies. Trial teams utilized radio programs, social media, SMS, and mobile outreach to disseminate information and capture community feedback in real time [41]. The use of transparent rumor registries facilitated systematic correction of misinformation, allowing emerging concerns such as fears about blood draws or vaccine side effects to be addressed promptly, thereby reducing barriers to recruitment and participation.

Community mistrust arose from perceptions that trials primarily benefited external actors, with minimal local gains [42]. To address this, researchers, sponsors, and national authorities established legally defined post-trial access plans and incorporated benefit-sharing measures, such as staff training, laboratory upgrades, and support for primary care [42]. These strategies fostered mutual partnership, reinforced community trust, and ensured that research outcomes translated into tangible local benefits.

## Discussion

This review highlights persistent structural, financial, and capacity-related barriers undermining effective community engagement (CE) in clinical trials across Africa. While CE is universally acknowledged as an ethical and scientific cornerstone, our findings reinforce earlier scholarship showing that CE remains poorly institutionalized, inconsistently funded, and inadequately professionalized, resulting in fragmented practices that fall short of meaningful engagement.

The overwhelming predominance of international donor funding estimated at 90% within the sub-Saharan African clinical trial sector fosters a state of financial precariousness that undermines long-term institutionalization [29]. Consequently, the conclusion of external funding often triggers the erosion of localized infrastructure, highlighting the urgent need for domestic resource mobilization to bridge the transition from project-based interventions to sustained research sovereignty.

### Chronic underfunding and consequences for meaningful engagement in clinical trials

Within the African clinical research ecosystem, CE is frequently reduced to a transactional logic where resources are prioritized for recruitment rather than relationship-building [5]. This review identifies a critical dependency cycle: the reliance on project-specific international donor funding ensures that CE remains an ephemeral trial component rather than a sustainable institutional capacity. This results in a recurring loss of operational continuity and community-based assets upon the cessation of external support. Systematic analysis suggests a prevailing conceptualization of CE as an ancillary task rather than a foundational research pillar [46]. This is underscored by the frequent absence of dedicated, ring-fenced budgetary allocations specifically for specialized personnel, logistical infrastructure, and linguistic translation services essential for robust, longitudinal engagement. The resultant reliance on informal volunteerism, opportunistic integration with existing public health frameworks, or transient project-specific funding cycles engenders a lack of procedural continuity and diminishes institutional accountability. Consequently, a transactional funding logic persists, wherein resources are prioritized for recruitment and data acquisition, frequently culminating in the “abandonment” of participant communities once enrollment metrics are achieved. This paradigm privileges short-term technical outputs over the sustained relationship-building and knowledge translation necessary to fulfil the ethical requirement of respect for communities [44,47]. Fragmentary community engagement (CE) strategies exacerbate historical mistrust and facilitate the dissemination of misinformation, reinforcing the perception of host communities as extractive data repositories rather than collaborative stakeholders [48]. This systemic under-resourcing is multifaceted, encompassing both fiscal constraints and a lack of strategic prioritization within the research ecosystem. Funding agencies frequently lack stringent mandates for CE implementation or oversight, and clinical trials rarely integrate validated CE metrics into formal reporting structures [5]. Consequently, the absence of standardized accountability mechanisms diminishes the institutional legitimacy of CE, entrenching a structural schism between research investigators and local populations [49].

### The role of funders in strengthening CE governance

The empirical evidence derived from the African Malaria Network Trust (AMANET) case studies necessitates a paradigm shift among funding bodies, moving from a conceptualization of community engagement (CE) as a discretionary adjunct to recognizing it as a fundamental ethical and methodological prerequisite [44]. These data demonstrate that when CE is structurally integrated into the longitudinal trial architecture through dedicated fiscal allocations for Community Advisory Boards (CABs) and context-sensitive deliberative mechanisms investigators observe significant optimizations in recruitment velocity and participant retention, alongside a marked attenuation of sociocultural misinformation [50]. To institutionalize these gains, it is imperative that funding agencies move beyond normative endorsements and implement structural mandates. Such mandates should require comprehensive CE strategies as a condition of award, encompassing the entire trial lifecycle—including post-trial dissemination and benefit-sharing—while simultaneously fostering durable infrastructure such as district-level engagement networks and standardized professional pathways for community-liaison personnel [51,52].

Furthermore, addressing the “implementation gap” identified in systematic reviews requires the integration of rigorous monitoring and evaluation (M&E) frameworks. The current dearth of standardized CE metrics precludes cross-trial benchmarking and diminishes institutional accountability; therefore, funders must require the adoption of validated indicators to evaluate engagement efficacy [52]. While these recommendations align with international normative benchmarks established by the Council for International Organizations of Medical Sciences (CIOMS) and the Good Participatory Practice (GPP) guidelines, the AMANET experience underscores that the transition from theory to practice is contingent upon the financial leverage and policy incentives uniquely held by the donor community [42,53].

### Training gaps and the absence of core competencies

A critical structural impediment identified across the literature is the pervasive deficit in standardized pedagogical frameworks for community engagement (CE) personnel. This lack of a structured competency matrix often results in role ambiguity and a perceived marginalization of CE staff within the investigative hierarchy. Consequently, engagement practitioners are forced to navigate high-stakes socio-political landscapes with insufficient preparation, leading to a precarious reliance on “mixed skill sets” that lack formal validation or professional career trajectories [54].

This systemic skills deficit generates significant downstream risks to trial integrity and ethical compliance. Inadequate training correlates with inconsistencies in the informed consent process and an institutional inability to effectively mitigate therapeutic misconceptions or epistemological distortions, such as localized rumors [55]. Furthermore, without advanced training in conflict resolution and qualitative feedback interpretation, CE staff may struggle to translate complex genomic or clinical concepts for lay audiences, thereby weakening the bidirectional flow of information essential for genuine partnership.

To address these vulnerabilities, there is an urgent requirement for the development of supranational competency frameworks localized to the African research ecosystem. These frameworks must prioritize “political and sociocultural literacy” alongside traditional clinical metrics to ensure that engagement is both scientifically rigorous and ethically sound [36]. Aligning these curricula with Good Participatory Practice (GPP) guidelines and continental research strategies is a necessary step toward establishing formal credentialing systems. Transforming CE from an administrative function into a recognized, specialized profession is essential for fostering institutional accountability and ensuring the long-term sustainability of clinical research in the region as required [52,53].

### Structural and operational barriers compromising CE quality

The literature consistently identifies systemic structural impediments that compromise the efficacy of community engagement (CE) in African clinical trials, primarily driven by truncated trial timelines, suboptimal regulatory frameworks, and entrenched historical mistrust. A primary driver of community skepticism is the prevalence of “open-loop” research cycles, wherein a lack of post-trial dissemination reinforces perceptions of epistemic extractivism, the view that communities are merely repositories for data harvesting rather than collaborative partners [36]. This issue is compounded by linguistic and cultural heterogeneity; the failure to translate complex scientific objectives into accessible, culturally resonant discourse effectively disenfranchises local populations and diminishes community ownership of research outcomes. Furthermore, the temporal misalignment of CE often initiated retroactively following protocol approval, precludes meaningful community contribution to study design, while hierarchical power differentials between primary investigators and CE practitioners marginalize engagement expertise throughout the implementation phase [30,50].

In order to mitigate these barriers, a transition toward sustainable, institutionalized engagement is required, predicated on comprehensive systemic reform. Central to this evolution is the integration of CE within national and regional research governance architectures, as exemplified by current Africa CDC initiatives to embed engagement protocols into clinical trial preparedness frameworks [56]. Professionalizing the CE workforce requires the establishment of regional training centers and standardized career pathways, underpinned by rigorous accreditation mechanisms and defined competency frameworks. Concomitantly, regulatory and ethical review bodies must mandate the inclusion of auditable CE indicators, ensuring that engagement performance is a measurable component of trial oversight [52]. Finally, shifting from volatile, project-based funding to longitudinal, multi-year fiscal models is essential to ensure the continuity of community partnerships beyond the lifecycle of individual trials, thereby fostering a research ecosystem built on transparency and mutual accountability [51].

### Strengths and limitations

This review’s major strength lies in its methodological transparency, utilizing the MMAT (2018) to evaluate the analytical quality of diverse evidence types. By systematically mapping over 25 years of CE practice against the IAP2 Spectrum, this study provides the first comprehensive political economy of CE funding and workforce capacity in Africa.

Despite our rigorous approach, several limitations persist. Firstly, the inclusion of only English and French studies likely excludes vital data from Lusophone (Portuguese-speaking) and Arabic-speaking African regions, creating a geographic blind spot. Secondly, the review is subject to Social Desirability Bias; researchers are incentivized to report successful engagement to satisfy donor requirements, leading to an underreporting of failed initiatives or community resistance. Moreover, the extreme sociocultural heterogeneity of the continent means that a Pan-African landscape is inherently an abstraction; for instance, localized power dynamics in a genomic trial in South Africa may not mirror those of a malaria trial in Mali. Finally, the absence of standardized metrics across the 30 studies precluded a formal meta-analysis, restricting our ability to definitively correlate CE modalities with quantitative trial outcomes.

## Conclusion

Strengthening community engagement (CE) in clinical trials has transitioned from a normative ethical preference to a strategic imperative for Africa’s health security, research sovereignty, and pandemic preparedness. This systematic review has demonstrated that despite its recognized value, CE remains chronically underfunded, structurally precarious, and professionally marginalized within the current trial ecosystem.

The evidence is unequivocal: trials that treat communities as partners rather than “extractive data repositories” achieve superior recruitment velocity, higher participant retention, and more robust ethical compliance. Conversely, the instrumentalization of CE using it merely to facilitate enrollment fuels historical mistrust and leaves trials vulnerable to delays and social resistance.

For global health funders and national governments, the path forward requires a shift from project-based volunteer models to institutionalized, ring-fenced financing. This includes the professionalization of the CE workforce through standardized career pathways and the integration of CE into national research governance architectures. In an era of expanding genomic studies and rapid vaccine development, Africa’s health security depends on an agile, community-anchored research system. By catalyzing this transformation, stakeholders can ensure that clinical innovation is not only scientifically rigorous but also socially grounded and ethically sustainable.

## Supporting information

S1 Data Spreadsheet of all studies identified in literature search excluding grey literature. (ZIP)

S2 Data Spreadsheet of all excluded studies including reasons for exclusion. (ZIP)

S3 Data Dataset of registered African clinical trials including funding organizations obtained from the NIH World RePORT. (ZIP)

S1 Table Table of numbered list of included studies. (DOCX)

S1 Checklist PRISMA checklist of study. Note: The PRISMA 2020 checklist is open access and distributed under the terms of the Creative Commons Attribution 4.0 International (CC BY 4.0) License (http://creativecommons.org/licenses/by/4.0/).
(DOCX)

S1 RScipt Rdata: R Script with codes for geospatial visualization. (R)

S1 File Annex 1. Methodological quality assessment of included studies using the MMAT (2018). (DOCX)

## Abbreviations

AMED: Japan Agency for Medical Research and Development
BMBF: Federal Ministry of Education and Research (Germany)
CDC: US Centers for Disease Control and Prevention
CIHR: Canadian Institutes of Health Research
EC: European Commission
EDCTP: European and Developing Countries Clinical Trials Partnership
GACD: Global Alliance for Chronic Disease
GF: Gates Foundation
MRC: Medical Research Council (UK)
NHMRC: National Health and Medical Research Council
NIH: National Institutes of Health
Pasteur: Pasteur Institute
SRC: Swedish Research Council
Wellcome: Wellcome Trust
