# Supplementary material for: The precarious state of community engagement in African clinical trials: A systematic review of funding, workforce gaps, and the path to institutionalization
Source: PLOS Glob Public Health. 2026 Aug 13;6(8):e0006484. doi: 10.1371/journal.pgph.0006484 (PMC13472408; doi:10.1371/journal.pgph.0006484)
Supplement: Supplementary file 1 [file pgph.0006484.s004.docx]

# Numbered list of included studies

| **Authors** | **Year of Publication** | **Title** | **Site** | **Study Design/Procedure** |
| --- | --- | --- | --- | --- |
| 1. Bain *et al.* | 2022 | Community engagement in research in sub-Saharan Africa: current practices, barriers, facilitators, ethical considerations and the role of gender - a  systematic review | Sub-Saharan Africa | Systematic Review |
| 2. Sangong *et al.* | 2025 | Effective community engagement in one health research in Sub-Saharan Africa: a systematic review | Sub-Saharan Africa | Systematic Review |
| 3. Pancras *et al*. | 2022 | The role of community advisory boards in community-based HIV clinical trials: a qualitative study from Tanzania | Tanzania | Qualitative Survey (In depth interviews and Focused Group Discussions) |
| 4. Mijumbi *et al.* | 2023 | Regulation of community  advisory boards during conduct of clinical trials in Uganda: a | Uganda | Qualitative Survey (Key Informant Interviews) |

|  |  | qualitative study involving  stakeholders |  |  |
| --- | --- | --- | --- | --- |
| 5. Lawrence et Stewart | 2016 | The Challenge of Community  Representation | Uganda | Qualitative Survey |
| 6. L Manda-Taylor | 2013 | Establishing community advisory boards for clinical trial research in Malawi: engendering ethical  conduct in research | Malawi | Interventional survey |
| 7. Baron *et al.* | 2018 | Collateral benefits: how the practical application of Good Participatory Practice can  strengthen HIV research in sub-  Saharan Africa | Sub-Saharan Africa | Interventional Study |
| 8. Folayan *et al.* | 2020 | Considerations for stakeholder engagement and COVID-19  related clinical trials’ conduct in  sub-Saharan Africa | Sub-Saharan Africa | Interventional Study |
| 9. Gobat *et al* | 2025 | Better engagement, better evidence: working in partnership with patients, the public, and  communities in clinical trials | Multi-national including  member | Literature Review |

|  |  | with involvement and good participatory practice | states of the African  Union |  |
| --- | --- | --- | --- | --- |
| 10. Ndembi *et al* | 2024 | Strengthening and expanding capacities in clinical trials: advancing pandemic prevention, preparedness and response in  Africa | Entire Africa | Interventional Study |
| 11. Graef *et al.* | 2020 | Operational Strategies for Clinical Trials in Africa | Entire Africa | Multicenter Study |
| 12. Africa CDC | 2023 | Meeting Report: Optimizing Efficiency and Impact in the  African Clinical Trials Ecosystem | Entire Africa | Meeting Report |
| 13. Marsh *et al.* | 2008 | Beginning community engagement at a busy biomedical research programme: Experiences from the KEMRI CGMRC-Wellcome Trust Research  Programme, Kilifi, Kenya | Kenya | Interventional study |
| 14. Nakibinge *et al.* | 2009 | Community engagement in health | Uganda | Review |

|  |  | research: two decades of experience from a research project  on HIV in rural Uganda | (Rural) |  |
| --- | --- | --- | --- | --- |
| 15. Reddy *et al.* | 2012 | The role of community advisory boards in health research: Divergent views in the South  African experience | South Africa | Mixed Study |
| 16. Nyika *et al*. | 2010 | Engaging diverse communities participating in clinical trials: case examples from across Africa | Burkina Faso, Mali, Tanzania | Interventional Study |
| 17. Folayan *et al.* | 2019 | Considerations for community engagement when conducting clinical trials during infectious disease emergencies in West Africa | West Africa | Interventional Study |

| 18. Newman *et al,* | 2015 | Towards a Science of Community Stakeholder Engagement in Biomedical HIV Prevention Trials: An Embedded Four-  Country Case Study | Thailand, India, South Africa and Canada. | Interventional Study |
| --- | --- | --- | --- | --- |
| 19. European and Developing Countries Clinical Trials Partnership | 2021 | The EDCTP eMagazine offering background information and broader topics regarding the  programme. | Africa | Descriptive on EDCTP funding |
| 20. Tindana *et al.* | 2020 | Ethical challenges in community engagement practices in research during the COVID-19 pandemic  in Africa | Africa | Literature Review |
| 21. Yakubu *et al.* | 2018 | Model framework for governance of genomic research and biobanking in Africa - a content description | Africa | Descriptive Study |
| 22. The Global Health Network | 2017 | Article: Community Planning Toolkit: Community Engagement  mesh | Africa | Grey Literature |

| 23. Pratt et de Vries | 2018 | Community engagement in global health research that advances  health equity | Sub-Saharan Africa | Descriptive study |
| --- | --- | --- | --- | --- |
| 24. Pratt *et al*. | 2020 | Solidarity and Community Engagement in Global Health  Research | Africa | Descriptive study |
| 25. Tindana *et al.* | 2011 | Aligning community engagement with traditional authority structures in global health research: a case study from  northern Ghana | Ghana | Descriptive study |
| 26. Clinical Trials Transformation Initiative (CTTI) | 2015 | Presenting CTTI Recommendations: Effective Engagement with Patient Groups  Around Clinical Trials | Africa | Gray Literature |
| 27. World Health Organization | 2018 | Risk Communication and Community Engagement Preparedness and Readiness Framework: Ebola Response in the Democratic Republic of the  Congo in North Kivu | Congo | Report (Gray Literature) |

| 28. Baker *et al*. | 2025 | ALERRT Capacity Strengthening Programme - knowledge brokering  across international networks to support co-development and enhance  regional research capabilities | Africa | Research Training Report |
| --- | --- | --- | --- | --- |
| 29. Asante *et al.* | 2013 | Community engagement in biomedical research in an African setting: the Kintampo Health Research Centre experience | Ghana | Interventional survey |
| 30. COUNCIL FOR INTERNATIONAL ORGANIZATIONS OF MEDICAL SCIENCES | 2016 | International Ethical Guidelines for  Health-related Research  Involving Humans | Global (Including Africa) | Grey Literature |
