# Supplementary material for: The precarious state of community engagement in African clinical trials: A systematic review of funding, workforce gaps, and the path to institutionalization
Source: PLOS Glob Public Health. 2026 Aug 13;6(8):e0006484. doi: 10.1371/journal.pgph.0006484 (PMC13472408; doi:10.1371/journal.pgph.0006484)
Supplement: Supplementary file 3 [file pgph.0006484.s007.docx]

# **Annex**

## **Annex 1 Methodological Quality Assessment of Included Studies Using the MMAT (2018)**

| **Study ID** | **Study Design** | **Screening Questions** | **Criterion 1** | **Criterion 2** | **Criterion 3** | **Criterion 4** | **Criterion 5** | **Overall Quality Ranking** |
| --- | --- | --- | --- | --- | --- | --- | --- | --- |
| Bain *et al.,* 2022 | Qualitative | Yes | Yes | Yes | Yes | Yes | Yes | *****High quality |
| Sangong *et al*., 2025 | Qualitative | Yes | Yes | Yes | Yes | Yes | Yes | *****High quality |
| Pancras *et al*., 2022 | Qualitative | Yes | Yes | Yes | Yes | Yes | Yes | *****High quality |
| Mijumbi *et al*., 2023 | Qualitative | Yes | Yes | Yes | Yes | Yes | Yes | *****High quality |
| Lawrence *et al*., 2016 | Qualitative | Yes | Yes | Yes | Yes | Yes | Can’t tell | ****Moderate-high quality |
| Manda-Taylor, 2013 | Qualitative | Yes | Yes | Yes | Yes | Yes | Yes | *****High quality |
| Baron *et al*., 2013 | Qualitative | Yes | Yes | Yes | Yes | Yes | Yes | *****High quality |
| Folayan *et al*., 2020 | Qualitative | Yes | Yes | Yes | Yes | Yes | Yes | *****High quality |
| Gobat *et al*., 2025 | Qualitative | Yes | Yes | No | No | Yes | No | **Low-moderate quality |
| Ndembi *et al*., 2024 | Qualitative | Yes | Yes | No | No | Yes | Can’t tell | **Low-moderate quality |
| Graef *et al*., 2020 | Qualitative | Yes | Yes | Can’t tell | Yes | Yes | Can’t tell | ***Moderate quality |
| Africa CDC, 2023 | Quantitative | Yes | Yes | Can’t tell | Can’t tell | No | Yes | **Low-moderate quality |
| Marsh *et al*., 2008 | Qualitative | Yes | Yes | Yes | Yes | Yes | Yes | *****High quality |
| Nakibinge *et al*., 2009 | Qualitative | Yes | Yes | No | No | Yes | Yes | ***Moderate quality |
| Reddy *et al*., 2012 | Qualitative | Yes | Yes | Yes | Yes | Yes | Yes | *****High quality |
| Nyika *et al*., 2010 | Qualitative | Yes | Yes | Can’t tell | Yes | Yes | Yes | ****Moderate-high quality |
| Folayan *et al*., 2019 | Qualitative | Yes | Yes | Can’t tell | Can’t tell | Can’t tell | Can’t tell | *Low quality |
| Newman *et al*., 2015 | Mixed | Yes | Yes | Yes | Yes | Yes | Yes | *****High quality |
| EDCTP, 2021 | Guide | Yes | Can’t tell | No | No | No | Yes | *Low quality |
| Tindana *et al*., 2020 | Qualitative | Yes | No | No | No | No | Yes | *Low quality |
| Yakubu *et al*., 2018 | Qualitative | Yes | No | No | No | No | Yes | *Low quality |
| The Global Health Network, 2017 | Qualitative | Yes | Yes | No | No | No | Yes | **Low-moderate quality |
| Pratt *et al*., 2018 | Qualitative | Yes | Yes | Can’t tell | Can’t tell | Yes | Yes | ***Moderate quality |
| Pratt *et al*., 2020 | Qualitative | Yes | Yes | No | No | Yes | Yes | ***Moderate quality |
| Tindana *et al*., 2011 | Qualitative | Yes | Yes | Yes | Yes | Yes | Yes | *****High quality |
| Clinical Trials Transformation Initiative, 2015 | Report | Yes | Yes | No | No | Yes | Yes | ***Moderate quality |
| W.H.O., 2018 | Operational Roadmap | Yes | Yes | No | No | Yes | Yes | ***Moderate quality |
| Baker *et al*., 2025 | Programme Report | Yes | Yes | No | No | No | Yes | **Low-moderate quality |
| Asante *et al*., 2013 | Qualitative | Yes | Yes | Yes | Yes | Yes | Yes | *****High quality |
| CIOMS, 2016 | Framework | Yes | Yes | No | No | Yes | Yes | ***Moderate quality |

***Note: MMAT = Mixed Methods Appraisal Tool.***

***Key***

***Overall Quality Rating (Scoring):***

***** (5 stars): High quality (all 5 criteria met).

**** (4 stars): Moderate-High quality (4 criteria met).

*** (3 stars): Moderate quality (3 criteria met).

** (2 stars): Low-Moderate quality (2 criteria met).

* (1 star): Low quality (1 criterion met).

0 stars: Very low quality (no criteria met).

***For Qualitative Studies (e.g., Example 1 & 5):***

1. Appropriate approach?
2. Adequate data collection?
3. Findings derived from data?
4. Interpretation substantiated by data?
5. Coherence between sources, collection, analysis, and interpretation?

***For Quantitative Non-randomized (e.g., Example 2):***

1. Representative participants?
2. Appropriate measurements?
3. Complete outcome data?
4. Confounders accounted for?
