# Supplementary material for: The precarious state of community engagement in African clinical trials: A systematic review of funding, workforce gaps, and the path to institutionalization
Source: PLOS Glob Public Health. 2026 Aug 13;6(8):e0006484. doi: 10.1371/journal.pgph.0006484 (PMC13472408; doi:10.1371/journal.pgph.0006484)
Supplement: Supplementary file 4 [file pgph.0006484.s009.docx]

# **Responses to Reviewer Comments**

**Response to Reviewer 1**

**Reviewer 1 Comment:** *In the abstract conclusion - it is mentioned that CE is important for vaccines - the earlier parts of the abstract mention clinical trials - does it follow that most clinical trials are in vaccines...*

**Response:** We thank the reviewer for pointing out this lack of explicit alignment in the early part of the abstract. The focus on vaccine sovereignty is indeed a central pillar of our study's rationale, driven by the Africa CDC’s strategic goal to produce 60% of vaccines locally by 2040.

To ensure the abstract's conclusion follows logically for the reader, we have revised the early section of the abstract to explicitly ground the review within this vaccine development context.

- **Abstract Revision:** We added the following contextual framing to the abstract background: *"In light of the Africa CDC’s mandate to achieve 60% local vaccine production by 2040, establishing robust community engagement (CE) frameworks within African clinical trials is vital."*
- **Main Text Clarification:** Furthermore, as suggested, we ensured that the descriptive summary of the 30 included studies explicitly notes the heavy concentration of infectious disease and vaccine-related trials within the analyzed literature, creating a seamless thread from introduction to conclusion.

**Response to Reviewer 2**

**Reviewer 2 Comment 1:** *The words "correlation" and "analysis" are somewhat out of place since there are no quantitative results given in the paper.*

**Response:** We completely agree with the reviewer's point. Given that this study utilizes a descriptive geospatial approach rather than formal statistical hypothesis testing, terms like "correlation" could be misleading. We have thoroughly reviewed the manuscript and replaced these instances with more precise terminology, such as "spatial distribution," "geospatial clustering," and "descriptive mapping," to accurately reflect the qualitative and descriptive nature of our findings.

**Reviewer 2 Comment 2:** *In the Materials and Methods section, authors should include a section on statistical analysis... List software... Describe technical details or procedures required to reproduce the analysis.*

**Response:** We appreciate this feedback and recognize the importance of methodological reproducibility for PLOS Global Public Health. In accordance with the journal's policy, we have added a dedicated **"Geospatial Visualization and Software"** subsection within the Materials and Methods section.

The added text reads as follows: *"****Geospatial Visualization and Software:*** *To evaluate the geographic distribution and funding architectures of the included clinical trials, descriptive geospatial mapping and spatial visualizations were executed using R statistical software (version 4.5.3; R Foundation for Statistical Computing) [15]. Country-level aggregate data extracted from the National Institutes of Health (NIH) World RePORT database were programmatically joined with a high-resolution administrative boundary shapefile of the African continent, retrieved from the Natural Earth database via the rnaturalearth package (v1.0.1). Spatial data geometries were managed and projected utilizing the Simple Features standard via the sf package.*

*To visually represent trial density, continuous choropleth maps were constructed using the ggplot2 framework. Color scales were mapped using the perceptually uniform and colorblind-friendly viridis palette to ensure visual accessibility and reduce bias in density interpretation. To facilitate comparative analysis across distinct funding organizations, multi-panel faceted maps were generated. Final figure dimensions, resolutions, and text scaling were systematically optimized to maintain legibility and graphic integrity across all panels. In accordance with open-science and reproducibility principles, the fully annotated R script, underlying spatial datasets, and source NIH World RePORT extraction files are hosted and publicly available in the Supporting Information.*
