# Supplementary material for: The precarious state of community engagement in African clinical trials: A systematic review of funding, workforce gaps, and the path to institutionalization
Source: PLOS Glob Public Health. 2026 Aug 13;6(8):e0006484. doi: 10.1371/journal.pgph.0006484 (PMC13472408; doi:10.1371/journal.pgph.0006484)
Supplement: Supplementary file 7 [file pgph.0006484.s003.zip › Codes for geospatial maps for african clinical trials.docx]

library(ggplot2)

library(sf)

library(rnaturalearth)

\# Load Africa Map

africa <- ne_countries(scale = "medium", continent = "africa", returnclass = "sf")

\# Merge with your funding data

\# ggplot(africa) + geom_sf(aes(fill = funding_level)) +

\# scale_fill_viridis_c(option = "plasma") + theme_minimal()
